# miR-615 facilitates porcine epidemic diarrhea virus replication by targeting *IRAK1* to inhibit type III interferon expression

**DOI:** 10.3389/fmicb.2022.1071394

**Published:** 2022-12-01

**Authors:** Hong-qing Zheng, Cheng Li, Xiao-fu Zhu, Wei-Xiao Wang, Bao-ying Yin, Wen-juan Zhang, Shu-lin Feng, Xun-hui Yin, He Huang, Yan-ming Zhang

**Affiliations:** ^1^Key Laboratory of Animal Epidemic Disease Diagnostic Laboratory of Molecular Biology in Xianyang City, Institute of Animal Husbandry and Veterinary Medicine, Xianyang Vocational Technical College, Xianyang, Shaanxi, China; ^2^Tianjin Institute of Animal Husbandry and Veterinary Medicine, Tianjin Academy of Agricultural Sciences, Tianjin, China; ^3^College of Veterinary Medicine, Northwest A&F University, Yangling, Shaanxi, China; ^4^Institute of Hemu Biotechnology, Beijing Hemu Biotechnology Co. Ltd., Beijing, China; ^5^Liangshan County Animal Husbandry and Veterinary Development Center, Liangshan County Animal Husbandry Bureau, Jining, China

**Keywords:** miR-615, IFN, innate immunity, porcine epidemic diarrhea virus, intestinal epithelial cells, miRNA high-throughput, NF-kappa B, *IRAK1*

## Abstract

Porcine epidemic diarrhea virus (PEDV) in the Coronavirus family is a highly contagious enteric pathogen in the swine industry, which has evolved mechanisms to evade host innate immune responses. The PEDV-mediated inhibition of interferons (IFNs) has been linked to the nuclear factor-kappa B (NF-κB) pathway. MicroRNAs (miRNAs) are involved in virus–host interactions and IFN-I regulation. However, the mechanism by which the PEDV regulates IFN during PEDV infection has not yet been investigated in its natural target cells. We here report a novel mechanism of viral immune escape involving miR-615, which was screened from a high-throughput sequencing library of porcine intestinal epithelial cells (IECs) infected with PEDV. PEDV infection altered the profiles of miRNAs and the activities of several pathways involved in innate immunity. Overexpression of miR-615 increased PEDV replication, inhibited IFN expression, downregulated the NF-κB pathway, and blocked p65 nuclear translocation. In contrast, knockdown of miR-615 enhanced IFN expression, suppressed PEDV replication, and activated the NF-κB pathway. We further determined that *IRAK1* is the target gene of miR-615 in IECs. Our findings show that miR-615 suppresses activation of the NF-κB pathway by suppressing the IRAK1 protein and reducing the generation of IFN-IIIs, which in turn facilitates PEDV infection in IECs. Moreover, miR-615 inhibited PEDV replication and NF-κB pathway activation in both IECs and MARC-145 cells. These findings support an important role for miR-615 in the innate immune regulation of PEDV infections and provide a novel perspective for developing new treatments.

## Introduction

The emerging and re-emerging Coronavirus family member porcine epidemic diarrhea virus (PEDV) attacks neonatal piglets and causedto cause acute watery diarrhea, with a substantial mortality rate. The global swine industry has sustained tremendous financial losses as a result of PEDV infections (Akira et al., 2006; Li et al., 2012; Dong and Soong, 2021). The target cells for PEDV are porcine intestinal epithelial cells (IECs), and PEDV strain CV777 could successfully infect an immortalized IEC line (Cao et al., 2015b; Lin et al., 2015; Wang et al., 2016). Previous studies have shown that type-I interferons (IFN-Is), which are produced by the host innate immune response, are crucial in limiting PEDV replication by eliciting an innate antiviral response (Zhang et al., 2016; Jegaskanda et al., 2018). IFN-Is also promote adaptive immunity during influenza virus infections by enhancing natural killer cell function (Hoffmann et al., 2015).

Type-III interferons (IFN-IIIs) are believed to employ the same antiviral mechanism as IFN-Is and induce IFN-stimulated gene expression, with IFN-III receptors being distributed primarily in gastrointestinal and respiratory epithelial cells (Li et al., 2016). Studies on PEDV-infected IECs (IPEC-J2), as well as several other reports, have shown that IFN-IIIs play an important role in inhibiting PEDV infection in IECs (Li et al., 2016; Lui et al., 2016). IFN-IIIs also exhibit strong antiviral activity in Vero cells (Ma et al., 2018; Xue et al., 2018). Therefore, IFN-IIIs may be key factors regulating PEDV infection.

Viruses generally develop diverse mechanisms to evade the host innate immune response, such as by antagonizing IFN production (Li et al., 2017a; Zhang et al., 2018b). IFN antagonism and production have also been implicated in PEDV mechanisms for evading innate immunity (Annamalai et al., 2015; Li et al., 2017b). Therefore, identification of the antiviral factors of the innate immune system is crucial for the control of PEDV infection. IRF3, nuclear factor kappa B (NF-κB), and IRF7 activation are crucial for the release of IFN-IIIs. IFN-λ1, −λ4, and -λ3 have been identified in IPEC-J2 cells (Li et al., 2017b; Zhang et al., 2018b). In small intestinal epithelial cells (IPEC-J2), PEDV inhibited IFN-III secretion through interfering with IRF and NF-κB (Zhang et al., 2018b). However, PEDV regulates small intestinal epithelial cells of different origins in different ways. In IECs, PEDV could induce NF-κB activation through the Toll-like receptor (TLR)2, TLR3, and TLR9 pathways in porcine intestinal epithelial cells at 24 h.

The NF-κB pathway has been reported to affect the secretion of IFN-IIIs more potently than IFN-I pathways (Pu et al., 2017). Additionally, the majority of TLRs use myeloid differentiation primary response 88 (MyD88), which participates in the recruitment of interleukin-1 receptor-associated kinase (IRAK)1 and 4 (Fisher et al., 2021). Tumor necrosis factor (TNF) receptor-associated factor 6 (TRAF-6) is triggered by IRAK1 phosphorylation, which activates NF-κB and mitogen-activated protein kinase (MAPK; Akira et al., 2006). Furthermore, PEDV-infected cells were found to alter the activity of the NF-κB pathway (Wang et al., 2016).

MicroRNAs (miRNAs) are small RNAs that are 18–23-nucleotides in length and exhibit various effects on cell proliferation, differentiation, and apoptosis, and in viral infections (Huang et al., 2018). Viral infections result in the dysregulated expression of miRNAs, and these changes in miRNA abundance can in turn affect viral infection and cellular physiological processes by regulating innate immunity. Several reports have shown that miRNAs inhibit PEDV infection by downregulating different target genes that are required for innate immunity (Wu et al., 2013; Zheng et al., 2018; Qi et al., 2021). In particular, miR-221-5p was found to decrease the rate of PEDV replication in MARC-145 cells by boosting activation of the NF-κB pathway (Zheng et al., 2018). In addition, miR-129-3p, which was identified in the process of studying porcine circovirus 2-infected cells, inhibited PEDV replication by targeting the NF-κB pathway in IPEC-J2 cells (Annamalai et al., 2015). By suppressing the expression of the proteins acting downstream of the NF-κB pathway in porcine kidney (PK) cells, miR-30c-5p reduces the expression of IFN-IIIs (Zhao et al., 2012; Buggele and Horvath, 2013; Song et al., 2015). However, the role and underlying mechanism of the miRNA-mediated regulation of the NF-κB pathway in PEDV infection in IECs have not yet been elucidated.

In this study, to ascertain the function of miRNAs in the innate immune response to PEDV infection, we performed high-throughput sequencing on PEDV-infected IECs, revealing a change in miRNA expression and innate immunity pathways under infectious conditions. Among the screened miRNAs, miR-615 was predicted to function in the NF-κB pathway and facilitate PEDV replication. We further found that miR-615 inhibits IFN-III expression and NF-κB pathway activation by targeting IRAK1. Conversely, miR-615 induced PEDV replication and inhibited the NF-κB pathway in two different types of cells (IECs and MARC-145). These data imply that miR-615 is a crucial PEDV target, and thus may be a potential target for PEDV treatment and prevention strategies.

## Materials and methods

### Cells and viruses

MARC-145 cells, which are kidney cells from an African green monkey, were grown in Dulbecco’s modified Eagle medium (DMEM; Hyclone, Logan, UT, USA) with 10% heat-inactivated fetal bovine serum (FBS; PAN-Biotech), and 100-times diluted penicillin and streptomycin (Hyclone). MARC-145 cells were used to validate the effect of miR-615 during PEDV infection. The IECs were cultivated in DMEM-F12 (Hyclone) supplemented with 10% FBS, penicillin, and streptomycin at the same concentrations as indicated above. All cells were kept in an incubator with 5% CO_2_ at 37°C as previously described (Wang et al., 2014). PEDV strains CV777 (GenBank accession number KT323979.1) and NW-17 (GenBank accession number MF782686.1) were provided by Nuoweilihua Biotechnology Co., which have an *S* gene from the epidemic strain group II. Strain NW-17 could infect IECs without trypsin and was therefore selected as an optimal strain for IEC infection. Vero cells were used to prepared the PEDV stock by three cycles of freezing and thawing, and were stored at −80°C.

### Immunofluorescence assays (IFAs)

After 20 min of fixation at a 4:1 ratio of cold acetone and methanol, the cell samples were washed three times in phosphate-buffered saline (PBS). The cells were incubated with a monoclonal antibody (mAb) against the PEDV N or NF-κB p65 protein for 2 h. The cells were then rinsed three times with PBS and incubated for 1 h with a fluorescein isothiocyanate-conjugated AffiniPure Goat Anti-rabbit/Mouse secondary antibody (Sungene Biotech, Tianjin, China). Hoechst 33258 was utilized to stain the nucleus, and the cell samples were analyzed with a laser-scanning confocal microscope after three PBS rinses (Olympus). The experiment was performed at room temperature.

### Mirna microarray and predicting the mRNA targets of differentially expressed miRNAs (DEMiRs)

Deep sequencing was carried out at Novogene (Beijing, China). IECs were infected in triplicates for 24 h with CV777, NW-17, or a mock infection at a multiplicity of infection (MOI) of 1. The mock group was treated with PBS. Total RNA was isolated using Trizol reagent (Invitrogen). Nine small RNA libraries (triplicate samples of the control mock-, CV777-, and NW-17-infected groups) were generated for Illumina sequencing. The microarray assay was conducted as previously described (Hallman et al., 2013). The prediction of target genes of DEMiRs was performed using miRanda (John et al., 2004; version 3.3a), PITA^1^, and RNAhybrid.^2^ Gene Ontology enrichment analysis was performed to screen the potential functions of the significantly enriched target genes. Kyoto Encyclopedia of Genes and Genomes (KEGG) pathway analysis was applied to identify the potential pathways associated with the target genes of the DEMiRs (Kanehisa et al., 2008). *p*-values were corrected using the Benjamini–Hochberg strategy (Benjamini and Hochberg, 1995). Statistical significance was defined as a corrected *p*-value <0.05.

### Validation of DEMiRs using reverse transcriptase-quantitative polymerase chain reaction (RT-qPCR)

Total RNA was extracted from IECs 24 h after infection at an MOI of 1. The Real-time Quantitative PCR Detection System and SYBR PrimeScript™ miRNA RT-PCR Kit were used to perform RT-qPCR. One microliter of each primer, 2.0 μl of diluted cDNA, and 12.5 μl of SYBR Green Premix Ex Taq II were included in each 25 μl reaction mixture. The thermocycling conditions comprised 95°C for 30 s, followed by 40 cycles of 95°C for 5 s and 60°C for 20 s. Each sample was processed in triplicates. Fifteen miRNAs were selected for testing. The expression of the U6 small RNA was used as the reference for data normalization. Table 1 contains the primer sequences for each miRNA and gene.

**Table 1:** Sequences of the miRNA mimic and inhibitor primers used in this study.

Genes	Forward primer (5′–3′)	Reverse primer (5′–3′)	Use
*susIFN-β*	AGTGCATCCTCCAAATCGCT	GCTCATGGAAAGAGCTGTGGT	RT-qPCR
g*PEDV*	AGTACGGGGCTCTAGTGCFigAG	GCTTATCCAAATTCTTCAGGCG	RT-qPCR
m*β-actin*	ATCGTGCGTGACATTAAG	ATTGCCAATGGTGATGAC	RT-qPCR
miR-615 mimics	UCCGAGCCUGGGUCUCCCUCU	AGAGGGAGACCCAGGCTCGGA	Overexpression of miR-615
miR-615 inhibitors	AGAGGGAGACCCAGGCTCGGA		Silencing of miR-615
*U6*	CTCGCTTCGGCAGCACA	AACGCTTCACGAATTTGCGT	RT-qPCR
*IFN-λ1*	GGTGCTGGCGACTGTGATG	GATTGGAACTGGCCCATGTG	RT-qPCR
*IFN-λ3*	ACTTGGCCCAGTTCAAGTCT	CATCCTTGGCCCTCTTGA	RT-qPCR
*susβ-actin*	AGGGTGTAATGGTGGGAATG	GCCGTGTTCAATGGGGTAT	RT-qPCR
miR-615	AGCCTGGGTCTCCCTCTAAA		RT-qPCR
miR-708-5p	AAGGAGCTTACAATCTAGCTGGG		RT-qPCR
miR-30e-5p	GGTGTAAACATCCTTGACTGGAAGCT		RT-qPCR
miR-148a-3p	TCAGTGCACTACAGAACTTTGT		RT-qPCR
miR-129a-5p	CTTTTTGCGGTCTGGGCTTG		RT-qPCR
miR-486	TTCCTGTACTGAGCTGCCC		RT-qPCR
miR-532-5p	CATGCCTTGAGTGTAGGACC		RT-qPCR
miR-542-3p	GGTGTGACAGATTGATAACTGAAA		RT-qPCR
miR-133a-5p	GAGCTGGTAAAATGGAACCAAAT		RT-qPCR
miR-206	TGGAATGTAAG GAAGTGTGTGA		RT-qPCR
miR-20a	ACTGCATTATGAGCACTTAAAG		RT-qPCR
miR-671-5p	AGGAAGCCCTGGAGGGGCTG		RT-qPCR

### Transfection of miRNA mimics, miRNA inhibitors, and siRNAs

A miRNA mimics is a chemically synthesized double-stranded RNA identical to a mature miRNA sequence. A miRNA inhibitor is a chemically modified single-stranded RNA that is complementary to the mature miRNA sequenc. After 12 h of culture, MARC-145 cells and IECs were transfected for 24 h using Lipofectamine 3000 (Invitrogen) with mimic control (MC), miR-615 mimics, miR-615 inhibitor (miR-615 inhi; 100 nM), inhibitor control (IC; 100 nM), small interfering RNA (siRNAs; si-IRAK1), or siRNA control (SC; 50 nM), which were synthesized at RiboBio (Guangzhou, China). Subsequently, the cells were infected with PEDV at an MOI of 1.0. After 24 h of infection, the cells were examined for indirect immunofluorescent labeling, or collected for RNA quantification or western blotting.

### Plasmids

pCDNA3.1+ plasmid was used to clone the Flag-IRAK1 plasmid at Genecreate using the BamHI and EcoRI restriction sites (Wuhan, China). The miRNA reporter plasmids, WT-pmirGLO-IRAK1 (wild-type, WT), and MuTpmirGLO-IRAK1 (mutant type; MuT) were subcloned into pmirGLO using the Nhel and SalI sites at Genecreate. pNiFty-luc plasmids is composed of a minimal Promoter, five NF-κB repeated transcription factor binding sites and a Luc (Luciferase) reporter gene of mammal.

### Dual-luciferase reporter assays

The potential miR-615 target genes in the 3′-untranslated region (3′-UTR) of IRAK1 were identified using the luciferase vector pmirGLO (Promega, Madison, WI, USA). The wild-type (WT-pmirGLO-IRAK1) or mutant (MuTpmirGLO-IRAK1) 3′-UTR sections of IRAK1 were subcloned into the pmirGLO vector and co-transfected into 293 T cells with miR-615. NF-κB activity was detected using pNiFty-luc and pRL-TK plasmids with the Dual-Luciferase Reporter Assay System (Promega, Madison, WI, USA) according to the manufacturer instructions.

### Western blotting

The cells were lysed using radioimmunoprecipitation assay buffer (Beyotime, Shanghai, China) and centrifuged at 4°C for 12,000 rpm. The concentration of the lysate was evaluated using a bicinchoninic acid (Thermo Scientific) protein assay kit. Each sample was diluted with 5Хloading buffer, boiled for 10 min, resolved on a sodium dodecyl sulfate-polyacrylamide gel electrophoresis gel, and then deposited onto polyvinylidene fluoride membranes at equal amounts. The membranes were blocked with 5% skim milk for 1 h and incubated overnight at 4°C with the primary antibodies against β-actin (1:1,000; 5,057; Cell Signaling Technology, Danvers, MA, USA), phospho-NF-κB p65 antibody (1:1,000; 3,033; Cell Signaling Technology), NF-κB p65 (1:1,000; 6,956; Cell Signaling Technology), and MyD88 (1:1,000; NB100-5698SS; RDSC). Proteins were detected using enhanced chemiluminescence detection reagents after being treated with secondary antibodies, HRP-conjugated goat anti-mouse IgG or goat anti-rabbit IgG (Beyotime). All samples were incubated along with β-actin as an internal standard. PEDV anti-nucleocapsid (N) protein antibody (1:1,000) was gifted by Prof. Tong Guangzhi, Shanghai Veterinary Research Institute.

### Statistical analyses

All data were statistically analyzed using Student’s t-test in Graphpad Prism 9.3.1 for analysis of variance (ANOVA).

## Results

### Dysregulated expression of miRNAs in circulating and vaccine strain-infected IECs

Villous epithelial cells are the primary target cells of PEDV infection; therefore, IECs are a suitable model for studying virus–cell interactions in the intestinal epithelia (Wang et al., 2014). IECs have been found to be susceptible to certain PEDV strains such as CV777, independent of high concentrations of trypsin (Cao et al., 2015b).

**Figure 1:**
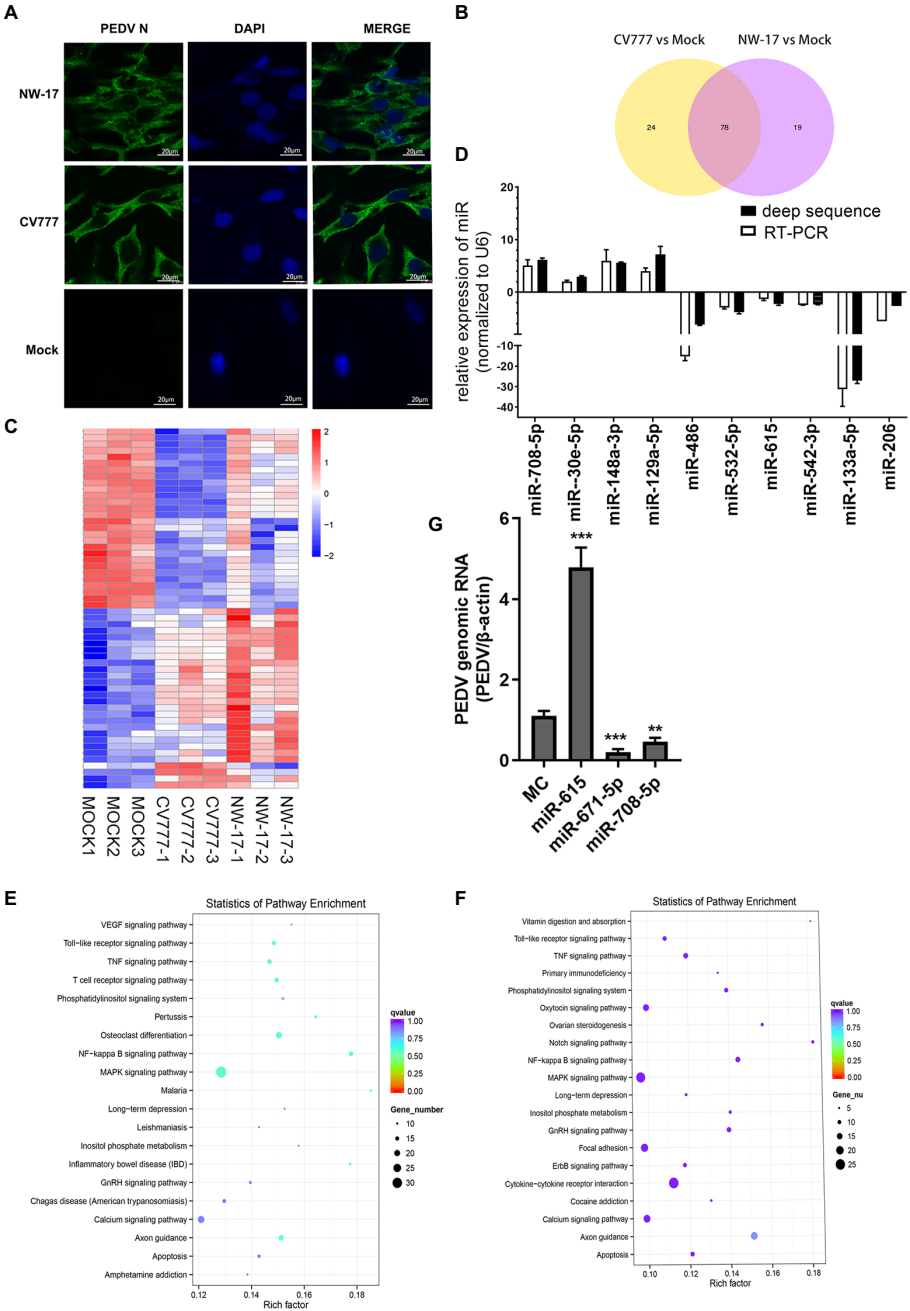
PEDV infection changes cellular microRNA profiles. **(A)** Immunofluorescence showing PEDV N protein expression in IECs (green). IECs were infected with the CV777 and NW-17 strains at a multiplicity of infection (MOI) of 1. Cells were fixed and stained with a mouse anti-N monoclonal antibody (mAb). Scale bar = 20 μm. **(B)** Venn diagrams showing the differentially expressed microRNAs (DEMiRs) among CV777-infected vs. mock-infected cells and NW-17-infected vs. mock-infected cells. **(C)** Heatmap showing the high abundance of DEMiRs. **(D)** Verification of the miRNA microarray assay using RT-qPCR. Data from RT-qPCR are shown as the mean ± SD of three independent experiments. U6 mRNA was detected as a control. (E and F) KEGG pathway enrichment analysis of the main DEMiRs for the CV777- and NW-17-infected groups, respectively. (G) Transfection of miRNAs inhibited or enhanced viral replication. IECs were transfected with the miR-671-5p, miR-708-5p, miR-615, or MC mimics at 50 nmol, followed by infection with PEDV (MOI = 1). Cells were collected for RT-qPCR at 24 h post-infection (hpi). PEDV genomic RNA was determined by RT-qPCR. Asterisks indicate statistical significance. *p < 0.05; **p < 0.01; ***p < 0.001.

The IFA results of the IECs at 24 h post-inoculation (hpi) with CV777 and NW-17 (MOI = 1) suggested significantly higher fluorescence in the CV777- and NW-17-infected groups than that in the mock-infected group (Figure 1A). This observation confirmed that CV777 and NW-17 could infect IECs effectively.

To identify whether miRNAs play a role in virus–cell interactions, we performed high-throughput sequencing to obtain the miRNA profiles of mock-infected or PEDV (CV777 and NW-17)-infected IECs at an MOI of 1 at 24 hpi. In comparison to the mock-infected group, 102 known miRNAs were found to be differentially expressed in the CV777-infected group and 98 known miRNAs were differentially expressed in the NW-17-infected group. Among them, 78 miRNAs were commonly differentially expressed in both infection groups (Figure 1B).

A total of 55 high-abundance miRNAs were selected by setting the read count to >250 (*p* < 0.05; Figure 1C). Of these miRNAs, 28 were upregulated and 27 were downregulated. To validate the high-throughput results of the DEMiRs, RT-qPCR was used to analyze the expression of 13 DEMiRs that were common to both groups (Figure 1D).

Using the KEGG pathway database, the roles of the DEMiRs in response to the PEDV strains were predicted, showing enrichment in several pathways, including the NF-κB, apoptosis, MAPK, TNF, and TLR signaling pathways, which participate in antiviral activities (Figures 1E,F). The high enrichment scores for the NF-κB and TLR signaling pathways indicated that innate immunity plays an important role in PEDV infection.

The DEMiRs enriched in the NF-κB pathway, miR-615, miR-708, miR-221-5p, and miR-671-5p, were selected for further investigation. Three miRNAs (miR-708, miR-671-5p, and miR-221-5p) were found to exert inhibition on PEDV replication by RT-qPCR. In particular, miR-615 overexpression significantly boosted viral replication compared to that in the MC group (*p* < 0.01; Figure 1G).

### Mir-615 promoted PEDV infection and replication in IECs and MARC-145 cells

**Figure 2:**
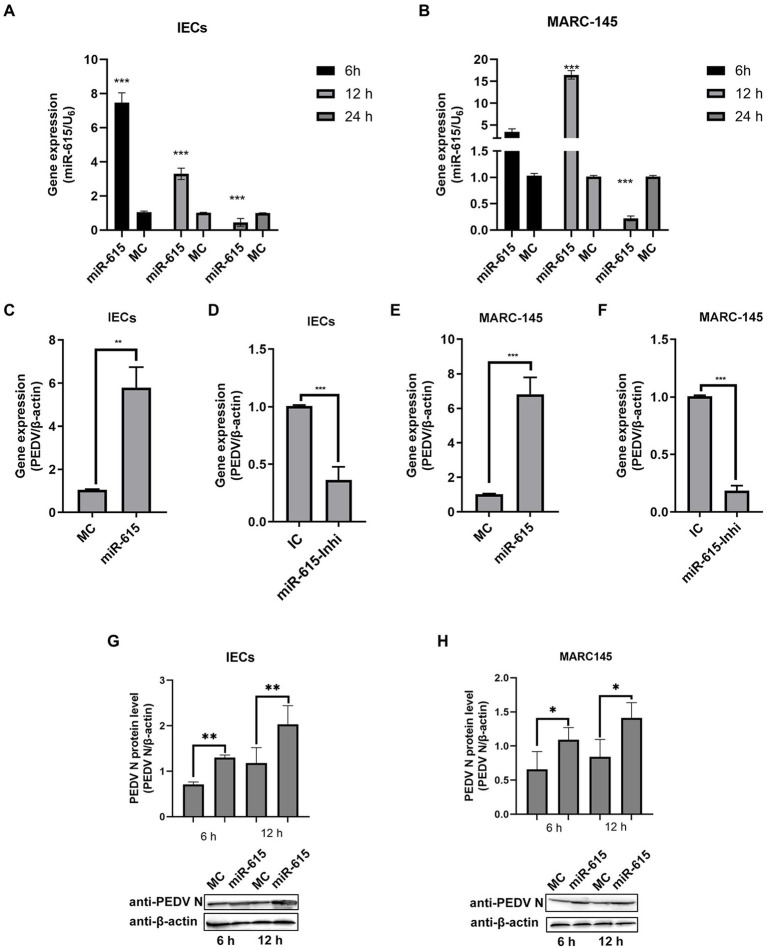
miR-615 facilitates PEDV infection in MARC-145 cells and IECs. (A and B) PEDV regulated miR-615 expression in IECs and MARC-145 cells. IECs **(A)** and MARC-145 cells **(B)** were infected with PEDV at a multiplicity of infection (MOI) of 1 and samples were collected at 6, 12, and 24 h post-infection. RT-qPCR was used to assess miR-615 expression. **(C–F)** IECs and MARC-145 cells were transfected with the MC, miR-615 mimic, IC, or miR-615 inhibitor (100 nmol). After 24 h, the cells were infected with PEDV at an MOI of 1. After 18 h, the PEDV genomic RNA of the MC, miR-615 mimic, IC, and miR-615 inhibitor in the IECs **(A,B)** and transfected MARC-145 cells **(C,D)** were assessed using RT-qPCR. PEDV N protein level was measured using western blotting following transfection with the MC or miR-615 mimic for 6 and 12 h in the IECs **(G)** and MARC-145 cells **(H)**. The intensity represents PEDV N protein levels normalized against that of β-actin across three independent experiments in IECs and MARC-145 cells, respectively. The data are presented as the mean ± SD of three independent experiments, performed with technical duplicates. *p<0.05, **p < 0.01, ***p < 0.001.

To further evaluate the interaction between miR-615 and PEDV in IECs and MARC-145 cells during PEDV infection, PEDV genomic and miR-615 RNA were detected using RT-qPCR. In IECs, the expression of miR-615 showed a downward trend (*p* < 0.01; Figure 2A). In MARC-145 cells, miR-615 expression was up-regulated at 12 h and was down-regulated at 24 h (*p* < 0.001; Figure 2B). The overexpression of miR-615 promoted viral replication in IECs (*p* < 0.01; Figure 2C) and MARC-145 cells (*p* < 0.001; Figure 2E). Conversely, the downregulation of miR-615 expression inhibited viral replication compared to that in the IC group in the IECs (*p* < 0.001; Figure 2D) and MARC-145 cells (*p* < 0.001; Figure 2F).

Western blotting showed that the level of PEDV N protein expression increased in IECs (Figure 2G) and MARC-145 cells (Figure 2H) after transfection with the miR-615 mimic for 6 and 12 h.

### Mir-615 inhibited the expression of IFN-IIIs in IECs and IFN-Is in MARC-145 cells

**Figure 3:**
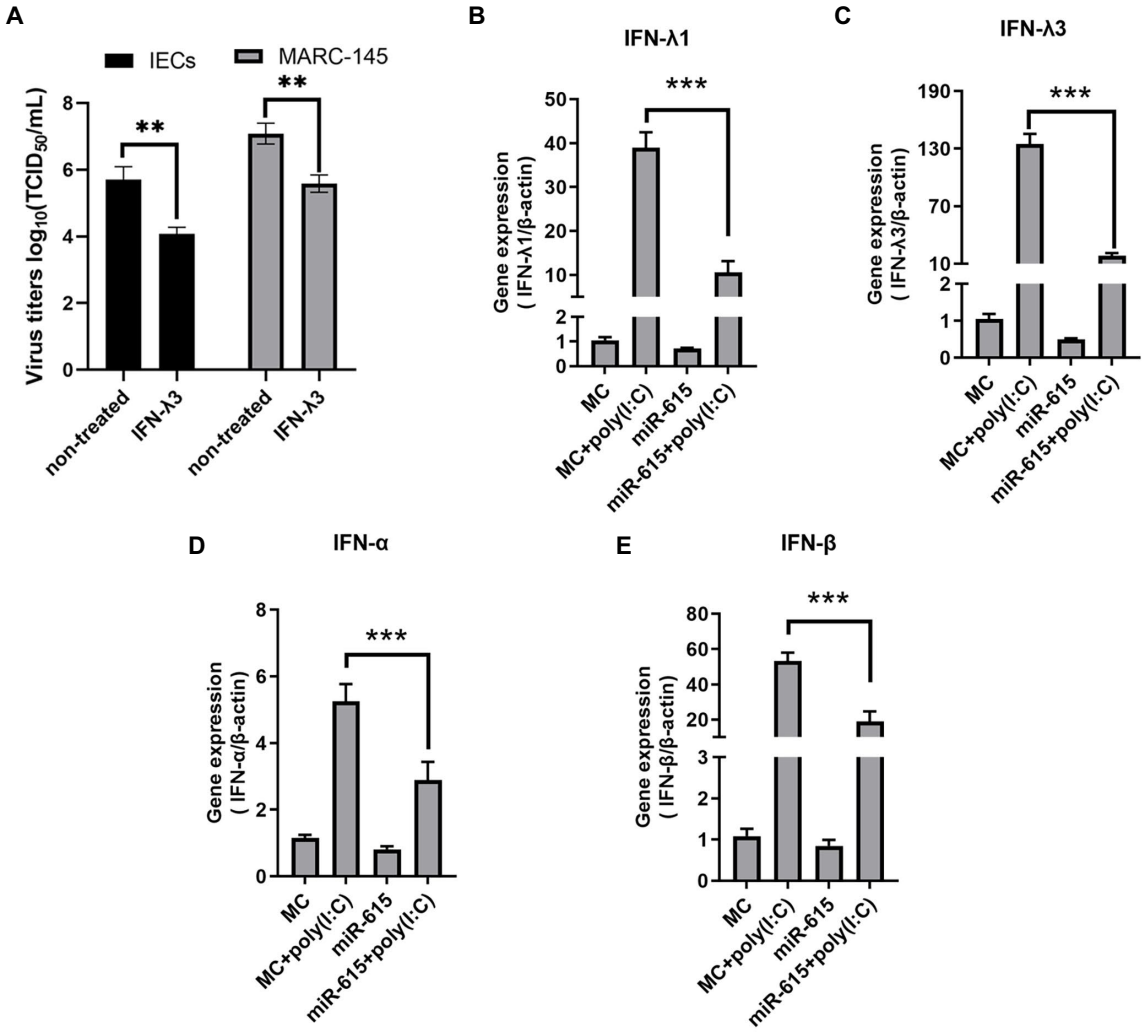
miR-615 downregulated the levels of IFN-Is in MARC-145 cells and of IFN-IIIs in IECs. **(A)** IFN-λ3 inhibited PEDV replication in IECs and MARC-145 cells. IECs and MARC-145 cells were seeded and treated with 100 ng/ml IFN-λ3 for 12 h, followed by infection with PEDV at a multiplicity of infection (MOI) of 1 with incubation for 2 h and replenished with fresh infection medium containing the IFN-λ3. Cell culture supernatants were collected at 12 and 24 h post-infection and titrated to determine the TCID_50_. MARC-145 and IECs were transfected with miR-615 for 24 h and then stimulated with poly (I:C; 10 μg/ml) for 24 h during PEDV infection. The cells were then harvested for RT-qPCR to determine the levels of **(B)** IFN-λ1 and **(C)** IFN-λ3 in the IECs, and of **(D)** IFN-α and **(E)** IFN-β in MARC-145 cells. The data are representative of three independent experiments (mean ± SD). ***p < 0.001, **p < 0.01.

Recent studies suggested that IFN-III has an important effect on the antiviral activity of small IECs (Zhang et al., 2018b). Consistently, we found that PEDV replication could be inhibited by IFN-λ3 (Figure 3A). Therefore, we hypothesized that miR-615 affects the expression of IFN-IIIs or IFN-Is, which in turn enhances viral replication. To test this hypothesis, we assessed the expression of IFN-IIIs in PEDV-infected IECs and of IFN-I in MARC-145 cells.

miR-615 mimics were transfected into the cells and/or poly (I:C) was used to stimulate the cells for 24 h. RT-qPCR was used to examine the expression of the two IFN-III subtypes, IFN-λ1 and-λ3. IFN-λ1 and λ3 expression was upregulated after stimulation with poly (I:C), which served as a positive control; however, the expression levels of IFN-λ1 and -λ3 significantly decreased following miR-615 transfection in IECs (*p* < 0.01; Figures 3B,C).

We also evaluated IFN-β and -α levels in miR-615-transfected MARC-145 cells. Poly (I:C) stimulation upregulated IFN-β and -α expression, whereas miR-615 transfection significantly downregulated their expression (*p* < 0.01; Figures 3D,E). The downregulation of IFN-Is and -III expression suggests that miR-615 may affect the IFN pathway in these two types of cells when infected with PEDV.

### Mir-615 inhibits NF-κB pathway activation in IECs and MARC-145 cells

**Figure 4:**
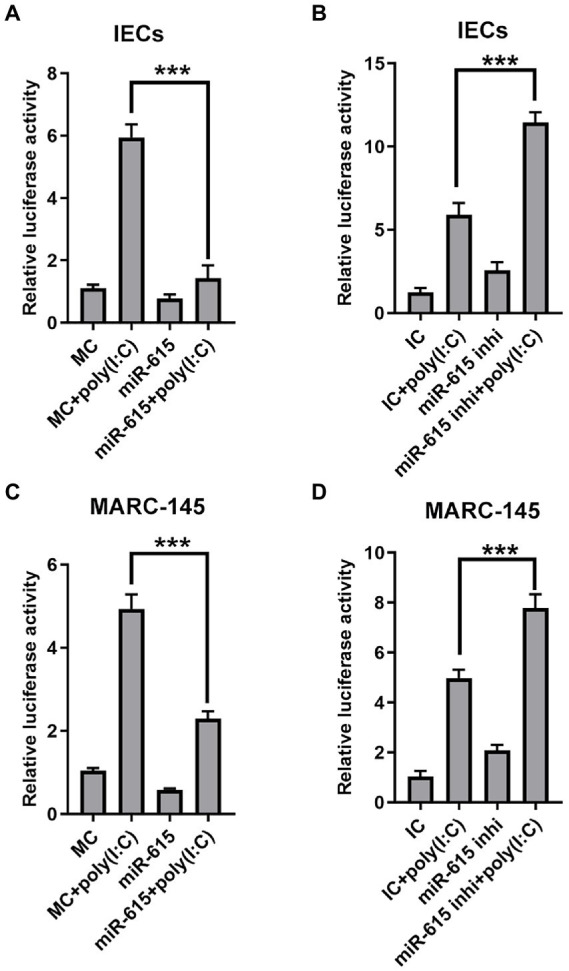
miR-615 inhibits NF-κB activation. Luciferase activity in IECs and MARC-145 cells. Cells were co-transfected using a dual-luciferase reporter system (pNiFty-luc and pRL-TK vectors, the activities of which indicate NF-κB-promoter activation) and miR-615 mimics or inhibitors for 24 h, and stimulated with poly (I:C) for 24 h during PEDV infection at a multiplicity of infection (MOI) of 1. Luciferase reporter activity of the miR-615 mimics in IECs **(A)** and MARC-145 cells **(C)**. Luciferase reporter activity of the miR-615 inhibitors in IECs **(B)** and MARC-145 cells **(D)**. The data are representative of three independent experiments (mean ± SD). ***p < 0.001.

Based on our preliminary findings, we hypothesized that miR-615 restricts the NF-κB pathway from being activated. The expression of IFNs is induced when NF-κB binds to the positive regulatory domain (PRD) II in the nucleus after translocation. Thus, we further examined NF-κB activity to explore whether the pathway was inhibited by miR-615 or miR-615 inhibitors. The NF-κB reporter luciferase plasmid (pNiFty-luc; containing five PRDII sites), thymidine kinase promoter-*Renilla* luciferase reporter plasmid (pRL-TK; an internal control plasmid), and miR-615 mimics or inhibitors were co-transfected into IECs, followed by poly (I:C) stimulation; thereafter, cell lysates were collected to detect luciferase activity at 24 hpi. The luciferase reporter assays showed that miR-615 markedly inhibited poly (I:C)-induced PRDII activity in IECs (*p* < 0.01; Figure 4A). Conversely, miR-615 inhibitors increased PRDII activity (*p* < 0.01; Figure 4B). Comparable results were observed in MARC-145 cells (Figures 4C,D). These results verified our hypothesis that miR-615 inhibits NF-κB pathway activity in both IECs and MARC-145 cells.

### Mir-615 inhibits NF-κB activation by suppressing p65 nuclear translocation

**Figure 5:**
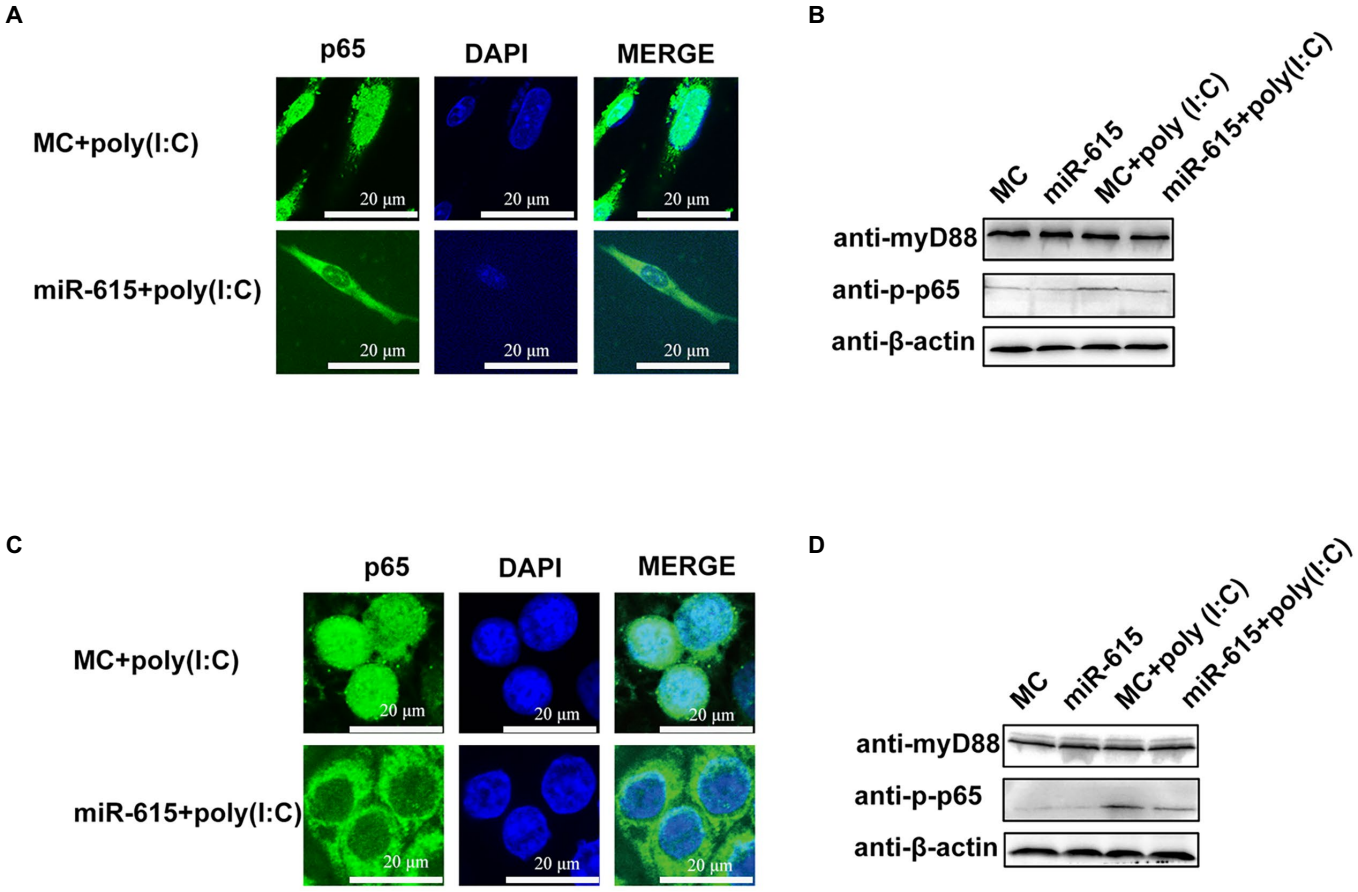
miR-615 inhibits the nuclear translocation and downregulates the phosphorylation of p65. **(A,C)** Immunofluorescence analysis of the nuclear translocation of p65 protein after transfection of miR-615 mimics during PEDV infection in IECs and MARC-145 cells. IECs and MARC-145 cells were transfected with miR-615. Poly (I:C) stimulation was added for 24 h during PEDV infection. Cells were fixed and stained with a rabbit anti-phosphorylated (p)-p65 and MyD88 monoclonal antibodies. Scale bar = 15 μm. **(B,D)** Western blot analysis for detection of the (p)-p65 and MyD88 proteins.

Next, we explored the mechanism of NF-κB inhibition. The IFA results revealed a significant reduction in the nuclear abundance of p65 protein in the miR-615 transfection group as compared to that in the MC-transfected group in both IECs (Figure 5A) and MARC-145 cells (Figure 5C). The abundance of p-p65 was found to decrease following transfection with miR-615 mimics in both the poly (I:C)-induced and non-poly (I:C)-induced groups (Figure 5B). We further examined p-p65 proteins upstream of MyD88 to investigate the mechanism underlying NF-κB downregulation. The results showed that p-p65 expression was downregulated, whereas MyD88 expression was not changed with the transfection of miR-615 in IECs (Figure 5B) and MARC-145 cells (Figure 5D). These results suggested that miR-615 inhibits the NF-κB pathway by repressing p65 nuclear translocation.

### Identification of *IRAK1* As a target gene of miR-615 in IECs

To explore the mechanism whereby miR-615 inhibits NF-κB activation, target gene prediction was conducted using miRanda^3^, PITA^4^, and RNAhybrid^5^, which identified 167 target genes (Supplementary Table S1). The most interesting candidate target gene was *IRAK1* as it had the highest prediction score (174) and was enriched in the NF-κB pathway. Therefore, we hypothesized that *IRAK1* is a target gene of miR-615.

**Figure 6:**
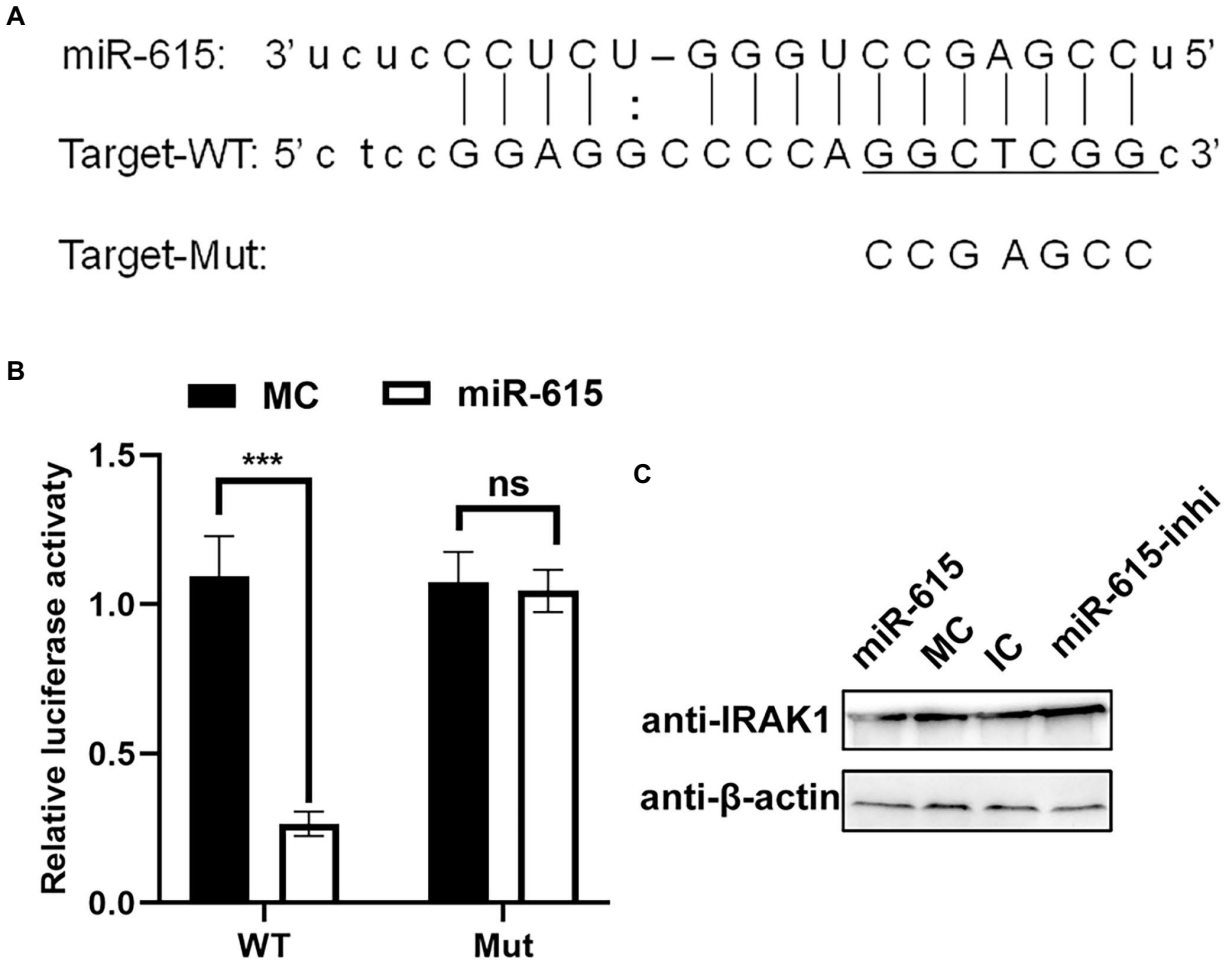
miR-615 targets *IRAK1.* **(A)** Bioinformatic prediction of the interactions between miR-615 and the 3′-UTR of swine *IRAK1*. For each schematic, the upper sequence is the sequence of mature miR-615, the middle sequence is the sequence in the binding site of miR-615 in the 3′-UTR of swine *IRAK1*, and the lower sequence is the mutated sequence of the *IRAK1* 3′-UTR. The seed sequence is underlined. **(B)** Luciferase activity in 293 T cells co-transfected with miR-615 mimics (or MC) and luciferase reporter gene plasmids containing the WT and Mut 3′-UTRs of *IRAK1* for 48 h. Data are normalized against firefly luciferase activity. Comparisons between groups were determined using Student’s t-tests. ***p < 0.001, **p < 0.01. **(C)** The level of the IRAK1 protein during transfection with miR-615 mimics or inhibitors in IECs detected using western blotting during PEDV infection. Western blotting was conducted using anti-IRAK1 antibody at 24 h post-infection (hpi).

Two reporter gene plasmids were constructed containing WT and seed region-Mut target sites (MuT) with matching or mutated target gene seed sites of miR-615 in the 3′-UTR of *IRAK1* (Figure 6A). miR-615 inhibited the luciferase activity of the WT reporter plasmid when compared with that in the MC-transfected group; however, miR-615 did not repress the activity of the mutated dual-luciferase reporter gene plasmid (Figure 6B). Moreover, transfection with miR-615 mimics downregulated IRAK1 protein expression, whereas transfection with miR-615 inhibitors upregulated its expression (Figure 6C).

### Mir-615 inhibits activation of the NF-κB pathway and promotes viral replication by targeting *IRAK1*

**Figure 7:**
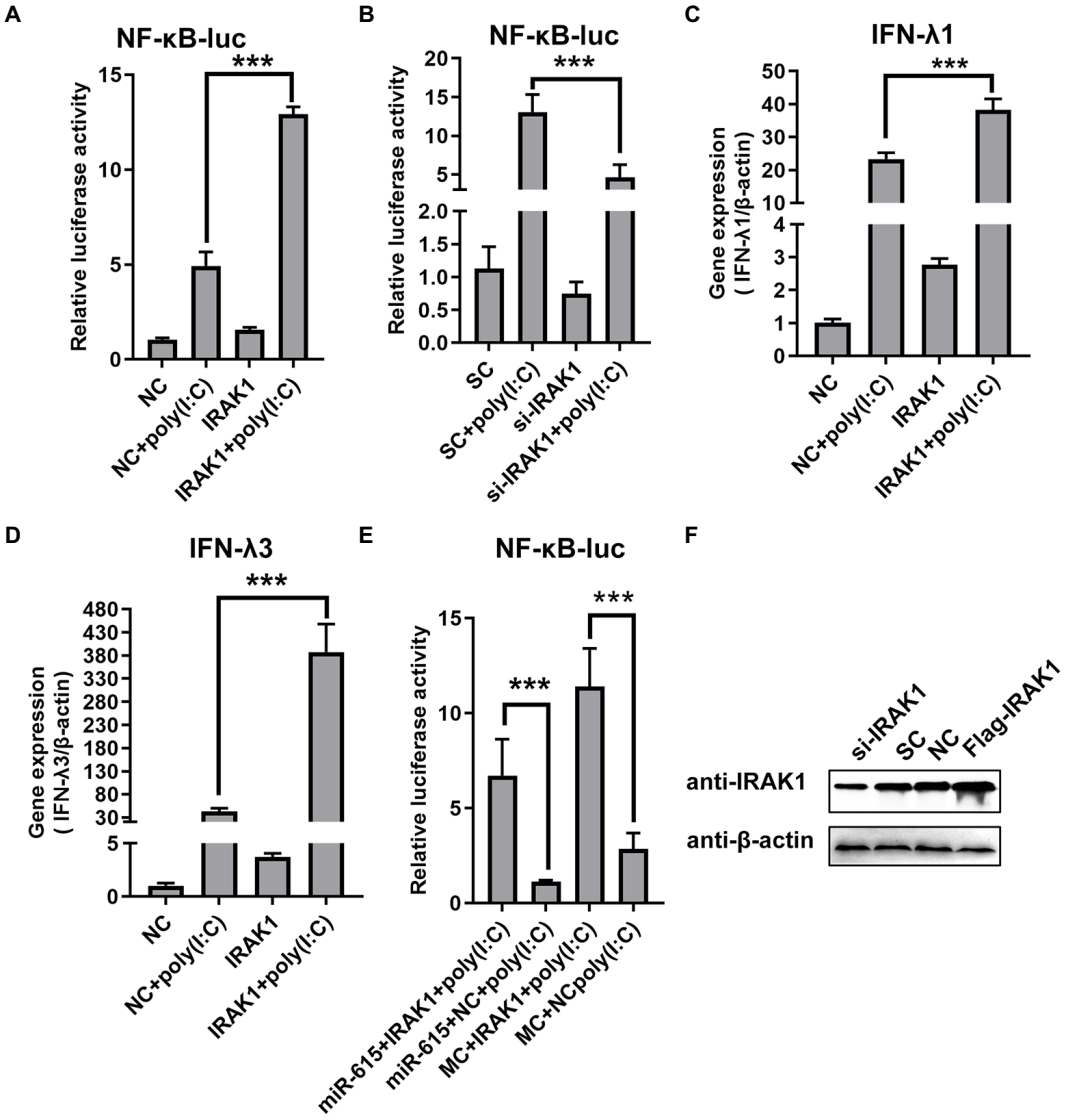
miR-615 inhibits NF-κB pathway activation by regulating *IRAK1*. **(A)** IRAK1-flag or negative control (NC) and **(B)** si-*IRAK1* or siRNA control (SC) were co-transfected with pNiFty-luc and pRL-TK into IECs. Cells were then stimulated with poly (I:C) and infected with PEDV (MOI = 1) at 24 h post-infection (hpi) or left untreated (unstimulated). Cells were then collected for the dual-luciferase reporter assay. **(C,D)** IECs were transfected with *IRAK1*-flag (or NC) or si-IRAK1 (or SC) for 24 h. Poly (I:C) stimulation and viral infection were then induced. After 24 h, IECs were collected for RT-qPCR to determine **(C)** IFN-λ1 and **(D)** IFN-λ3 expression. **(E)** Rescue experiment. Luciferase activity was rescued by *IRAK1* overexpression. IECs were co-transfected with miR-615 (or MC) and *IRAK1*-flag (or NC). At 24 hpi, poly (I:C) stimulation and viral infection were induced. After 24 h of stimulation, cells were collected and the luminescence activity was determined. **(F)** IRAK1 knockdown and overexpression were confirmed using western blotting. The data are representative of three independent experiments (mean ± SD). ***p < 0.001.

To determine whether repression of the NF-κB pathway by miR-615 is dependent on the regulation of *IRAK1* expression, we first examined the effect of IRAK1 on the NF-κB pathway using a dual-luciferase reporter assay with overexpression and knockdown of *IRAK1* in PEDV-infected IECs stimulated with poly (I:C). The NF-κB pathway was activated by *IRAK1* overexpression (Figure 7A) and was downregulated by knockdown of *IRAK1* (Figure 7B). In addition, *IRAK1* overexpression upregulated IFN-λ1 and -λ3 expression during viral infection (Figures 7C,D).

To further examine whether the effect of miR-615 on the NF-κB pathway was dependent on its regulation of *IRAK1* expression, miR-615 and *IRAK1* were overexpressed and the effects on the NF-κB pathway activity were examined. Porcine IECs were co-transfected with pNif-TK, pRL-TK, miR-615 mimics, or Flag-IRAK1, and poly (I:C) was used to induce NF-κB pathway activation. The rescue experiment showed that IRAK1 overexpression promoted NF-κB pathway activation and reversed the miR-615-dependent repression of the NF-κB pathway (Figure 7E). Collectively, these data indicate that miR-615 inhibits the NF-κB pathway by targeting *IRAK1*. The knockdown and overexpression of IRAK1 protein were further confirmed using western blotting (Figure 7F).

## Discussion

PEDV infection has been reported to antagonize innate immunity (Annamalai et al., 2015; Cao et al., 2015a). The main factors involved in antiviral innate immunity are IFN-Is and -IIIs. Notably, IFN-IIIs are involved in innate immunity in IECs. Multiple PEDV-encoded proteins inhibit the production of IFNs (Ding et al., 2014; Zhang et al., 2017). miRNAs have been reported to play a pivotal role in the regulation of viral infections by targeting the viral genome or regulating host cytokines to modulate the cellular environment (Bartel, 2009). To further examine the mechanisms and interactions between miRNAs and PEDV in porcine IECs, deep-sequencing techniques were used to analyze miRNA expression profiles. We found that PEDV infection altered cellular miRNA expression profiles in IECs. Furthermore, the innate immunity pathway was confirmed to be involved in PEDV infection. miR-615 was enriched in this pathway, and its overexpression promoted viral replication by inhibiting IFN-III expression and targeting IRAK1 in the NF-κB pathway. To the best of our knowledge, this is the first report of an miRNA targeting *IRAK1* during PEDV infection in IECs to function as a negative regulator of IFN-IIIs production.

PEDV infection has previously been shown to affect miRNA profiles and innate immunity (Huang et al., 2016; Zhang et al., 2018a; Qi et al., 2021). High-throughput sequencing of miRNAs in PEDV-infected IPEC-J2 cells revealed few identical differential miRNAs were expressed compared to our study. However, the KEGG pathway analysis showed that TLRs, Janus kinase-signal transducer and activator of transcription (JAK–STAT), retinoic acid-inducible gene I (RIG-I), and autophagy were involved in the response to PEDV infection. Our deep-sequencing KEGG analysis showed enrichment in the primary pathways involved in innate immunity (NF-κB and TLR signaling pathways; Zhang et al., 2018a). Another study using miRNA-mRNA high-throughput sequencing in PEDV-infected ST cells showed that innate immunity pathways were enriched following PEDV infection. Moreover, infection with different strains can induce the activation of different signaling pathways (Zhang et al., 2021). In PEDV-infected PK cells, high-throughput sequencing revealed few similar DEmiRNAs compared to those identified in other studies (Huang et al., 2016). However, PEDV-infected IECs (Cao et al., 2015b) showed activation of the NF-κB pathway. This may suggest that cells from the same source will have similar immune regulatory mechanisms after being infected by different PEDV strains. Our results thus provide insight into the mechanism of NF-κB pathway activation after PEDV infection of IECs. The inconsistency between our results and those of other studies may be attributed to different mechanisms of PEDV infection regulation in cells of different origins and the effects of different PEDV strains. Thus, our sequencing results provide insight on PEDV infection at the RNA level.

IFN-IIIs play a key role in antiviral innate immunity in the gut and at the mucosal surface. Compared with IFN-Is, IFN-IIIs preferentially inhibit PEDV infection in IECs (Li et al., 2017b). The robust activation of JAK–STAT signaling is induced to a greater degree by IFN-λ3 than by IFN-α. IFN-λ3 further plays a critical role in PEDV infection (Li et al., 2019). Similarly, IFN-λ1 exhibited strong anti-PEDV effects on IECs by activating the JAK–STAT signaling pathway. Furthermore, both IRF1 and NF-κB are related to PEDV-mediated IFN-IIIs suppression (Xue et al., 2018). In this study, we found that miR-615 downregulated IFN-λ1 and -λ3 expression by repressing the NF-κB pathway to facilitate PEDV replication. These results provide new insight into the mechanism by which IECs and PEDV interact through the NF-κB pathway. In addition, the transcription of IFN-III genes is more dependent on the NF-κB pathway than on the IRF system (Zhang et al., 2018b). This suggests that miR-615 is an important factor in cellular antiviral responses and an important anti-PEDV target. Notably, we did not identify changes in IFN-λ4 in IECs, which may be attributable to the low expression of IFN-λ4.

The inhibitory effect of miR-615 on the NF-κB pathway has been suggested in many other studies. In non-small cell lung cancer cells, miR-615-3p has been shown to be crucial in preventing cancer cell proliferation and metastasis by targeting insulin-like growth factor 2 (Liu et al., 2018). In breast cancer research, miR-615 was reported as a potential anti-onco-miR by targeting AKT serine/threonine kinase 2 expression (Bai et al., 2015). However, both the insulin-like growth factor 2 and AKT serine pathways could have an effect on the activity of the NF-κB pathway. In our study, miR-615 inhibited activation of the NF-κB pathway. This further suggests that miR-615 could exert its biological function by affecting the NF-κB pathway. It is worth noting that the NF-κB pathway is often activated in certain tumor cells, which further supports the role of miR-615 in affecting the NF-κB pathway. Finally, miR-615 promoted viral replication and inhibited the activation of the NF-κB pathway in both IECs and MARC-145 cells, suggesting the conserved role of miRNAs in different cells.

IRAK1 plays a critical role in TNF-α-induced NF-ĸB activation (Kim et al., 2012). The miRNAs miR-21, miR-146, miR-223, and miR-142a-3p have been reported to repress the NF-κB pathway by targeting *IRAK1* (Chen et al., 2013; Hung et al., 2013; Xu et al., 2013). In this study, we strongly suggest that miR-615 inhibited activation of the NF-κB pathway by targeting *IRAK1*. This further demonstrated the key role of IRAK1 in NF-κB pathway activation and also supports that the same target gene can be regulated by multiple miRNAs.

Collectively, our study showed that PEDV infection affects the NF-κB pathway and other innate immune-related pathways by changing the miRNA profiles. Our data further revealed the mechanism by which PEDV infection inhibits the secretion of IFN-IIIs in IECs and provides a new perspective for understanding the function of miR-615. Furthermore, we provide novel information regarding the intricate interplay between PEDV and cellular innate immunity in IECs during PEDV infection, which may offer new targets for the development of effective therapies to control PEDV and other coronaviruses.

## Data availability statement

The datasets presented in this study can be found in online repositories. The names of the repository/repositories and accession number(s) can be found in the article/Supplementary material.

## Author contributions

Y-MZ and HH designed the study. H-QZ, X-FZ, W-XW, and CL performed the experiments and collected the data. B-YY, W-JZ, S-LF, and X-HY analyzed and interpreted the data. H-QZ and X-FZ wrote the draft of the manuscript. W-XW, S-LF, B-YY, and CL edited and revised the manuscript. Y-MZ and HH coordinated the whole project. All authors contributed to the article and approved the submitted version.

## Funding

This research was funded in part by the Natural Science Basic Research Program of Shaanxi Province (grant number 2021JQ-900), Shaanxi Province Key Research and Development Project (grant number 2021NY-037), Research Fund Project of Xianyang Vocational and Technical College (2020KJA01 and 2018KYB01), Doctoral Research Foundation Project of Xianyang Vocational and Technical College (2021BK04 and 2019BK03), Key Research Projects of Xianyang Science, Technology Research and Development Program (grant number 2020 k02-63), and Innovative Research and Experimental Project of Young Scientific Researchers (grant number 2022012).

## Conflict of interest

Authors W-XW and HH were employed by Beijing Hemu Biotechnology Co., Ltd.

The remaining authors declare that the research was conducted in the absence of any commercial or financial relationships that could be construed as a potential conflict of interest.

## Publisher’s note

All claims expressed in this article are solely those of the authors and do not necessarily represent those of their affiliated organizations, or those of the publisher, the editors and the reviewers. Any product that may be evaluated in this article, or claim that may be made by its manufacturer, is not guaranteed or endorsed by the publisher.

## Footnotes

^1^ http://www.pita.org.fj/

^2^ https://bibiserv.cebitec.unibielefeld.de/rnahybrid/

^3^ http://www.microrna.org/microrna/hom.do

^4^ http://genie.weizmann.ac.il/pubs/mir07/mir07_dyn_data.html

^5^ https://bibiserv.cebitec.unibielefeld.de/rnahybrid/
