# Supplementary material for: miR-615 facilitates porcine epidemic diarrhea virus replication by targeting IRAK1 to inhibit type III interferon expression
Source: Front Microbiol. 2022 Dec 1;13:1071394. doi: 10.3389/fmicb.2022.1071394 (PMC9832332; doi:10.3389/fmicb.2022.1071394)
Supplement: Supplementary file 1 [file Table_1.DOCX]

Supplementary Material

# Supplementary Tables

Table 1S The target genes prediction of miR-615

| miRNA名称miRNA name | 靶基因  Target genes | 评分Score | 能量值Energy | 基因名字  Gene name |
| --- | --- | --- | --- | --- |
| ssc-miR-615 | ENSSSCG00000029460 | 141 | -22.23 | RND2 |
| ssc-miR-615 | ENSSSCG00000029570 | 153 | -32.85 | - |
| ssc-miR-615 | ENSSSCG00000029590 | 160 | -28.85 | ACD |
| ssc-miR-615 | ENSSSCG00000029761 | 150 | -25.02 | - |
| ssc-miR-615 | ENSSSCG00000029803 | 154 | -24.56 | KDM2B |
| ssc-miR-615 | ENSSSCG00000029877 | 146 | -19.7 | MKRN2 |
| ssc-miR-615 | ENSSSCG00000030155 | 145 | -20.64 | - |
| ssc-miR-615 | ENSSSCG00000030209 | 145 | -24.88 | MFNG |
| ssc-miR-615 | ENSSSCG00000030378 | 155 | -22.3 | LIMK1 |
| ssc-miR-615 | ENSSSCG00000030526 | 162 | -29.17 | - |
| ssc-miR-615 | ENSSSCG00000030805 | 146 | -20.18 | CH242-289B8.1 |
| ssc-miR-615 | ENSSSCG00000030888 | 151 | -28.88 | FADD |
| ssc-miR-615 | ENSSSCG00000030921 | 172 | -33.82 | APOA1 |
| ssc-miR-615 | ENSSSCG00000027404 | 155 | -20.89 | - |
| ssc-miR-615 | ENSSSCG00000027536 | 159 | -26.23 | - |
| ssc-miR-615 | ENSSSCG00000027750 | 145 | -20.64 | - |
| ssc-miR-615 | ENSSSCG00000027779 | 153 | -34.26 | TMEM259 |
| ssc-miR-615 | ENSSSCG00000027956 | 153 | -24.89 | SSH3 |
| ssc-miR-615 | ENSSSCG00000028049 | 153 | -33.91 | - |
| ssc-miR-615 | ENSSSCG00000028210 | 150 | -24.27 | NT5C |
| ssc-miR-615 | ENSSSCG00000028490 | 148 | -24.48 | - |
| ssc-miR-615 | ENSSSCG00000028755 | 154 | -22.02 | TSSC1 |
| ssc-miR-615 | ENSSSCG00000029135 | 146 | -20.18 | TNFRSF25 |
| ssc-miR-615 | ENSSSCG00000029161 | 148 | -21.2 | ADI1 |
| ssc-miR-615 | ENSSSCG00000026850 | 157 | -22.96 | - |
| ssc-miR-615 | ENSSSCG00000026857 | 159 | -27.32 | - |
| ssc-miR-615 | ENSSSCG00000027195 | 162 | -31.03 | - |
| ssc-miR-615 | ENSSSCG00000026403 | 151 | -23.97 | - |
| ssc-miR-615 | ENSSSCG00000026414 | 141 | -18.89 | ATOX1 |
| ssc-miR-615 | ENSSSCG00000025996 | 155 | -25.01 | - |

Table 1S (continued) The target genes prediction of miR-615

| miRNA名称miRNA name | 靶基因  Target genes | 评分Score | 能量值Energy | 基因名字  Gene name |
| --- | --- | --- | --- | --- |
| ssc-miR-615 | ENSSSCG00000017472 | 144 | -24.05 | IGFBP4 |
| ssc-miR-615 | ENSSSCG00000017499 | 155 | -25.01 | STARD3 |
| ssc-miR-615 | ENSSSCG00000017865 | 157 | -26.49 | CTNS |
| ssc-miR-615 | ENSSSCG00000017933 | 147 | -20.91 | - |
| ssc-miR-615 | ENSSSCG00000017986 | 141 | -19.8 | - |
| ssc-miR-615 | ENSSSCG00000018019 | 146 | -21.55 | - |
| ssc-miR-615 | ENSSSCG00000020690 | 155 | -22.59 | - |
| ssc-miR-615 | ENSSSCG00000020737 | 157 | -27.05 | - |
| ssc-miR-615 | ENSSSCG00000021186 | 155 | -22.74 | CRP |
| ssc-miR-615 | ENSSSCG00000021402 | 145 | -21.16 | - |
| ssc-miR-615 | ENSSSCG00000021418 | 163 | -32.93 | - |
| ssc-miR-615 | ENSSSCG00000021707 | 149 | -21.75 | ADCY7 |
| ssc-miR-615 | ENSSSCG00000021861 | 141 | -18.42 | - |
| ssc-miR-615 | ENSSSCG00000021865 | 151 | -20.64 | INHBA |
| ssc-miR-615 | ENSSSCG00000021880 | 147 | -21.28 | MXRA8 |
| ssc-miR-615 | ENSSSCG00000021967 | 151 | -30.07 | SYK |
| ssc-miR-615 | ENSSSCG00000022033 | 154 | -23.35 | SLC46A1 |
| ssc-miR-615 | ENSSSCG00000022614 | 149 | -22.26 | - |
| ssc-miR-615 | ENSSSCG00000022722 | 155 | -28.73 | - |
| ssc-miR-615 | ENSSSCG00000023078 | 147 | -20.75 | WDR4 |
| ssc-miR-615 | ENSSSCG00000023157 | 145 | -21.33 | HAGH |
| ssc-miR-615 | ENSSSCG00000023272 | 154 | -24.11 | ILK |
| ssc-miR-615 | ENSSSCG00000023423 | 153 | -23.17 | UBALD2 |
| ssc-miR-615 | ENSSSCG00000023478 | 154 | -23.35 | - |
| ssc-miR-615 | ENSSSCG00000023529 | 146 | -21.09 | - |
| ssc-miR-615 | ENSSSCG00000023594 | 142 | -19.57 | - |
| ssc-miR-615 | ENSSSCG00000023920 | 153 | -26.2 | - |
| ssc-miR-615 | ENSSSCG00000023994 | 158 | -27.77 | - |
| ssc-miR-615 | ENSSSCG00000024014 | 164 | -26.02 | - |
| ssc-miR-615 | ENSSSCG00000026182 | 157 | -25.88 | - |

Table 1S (continued) The target genes prediction of miR-615

| miRNA名称miRNA name | 靶基因  Target genes | 评分Score | 能量值Energy | 基因名字  Gene name |
| --- | --- | --- | --- | --- |
| ssc-miR-615 | ENSSSCG00000010254 | 149 | -24.14 | TACR2 |
| ssc-miR-615 | ENSSSCG00000010587 | 147 | -22.31 | TRIM8 |
| ssc-miR-615 | ENSSSCG00000010704 | 147 | -24.82 | LRFN1 |
| ssc-miR-615 | ENSSSCG00000010718 | 162 | -31.02 | CH242-129O9.1 |
| ssc-miR-615 | ENSSSCG00000011163 | 148 | -23.06 | - |
| ssc-miR-615 | ENSSSCG00000011322 | 151 | -25.45 | CCR1 |
| ssc-miR-615 | ENSSSCG00000011386 | 146 | -25.68 | MST1 |
| ssc-miR-615 | ENSSSCG00000011556 | 172 | -35.97 | IL17RE |
| ssc-miR-615 | ENSSSCG00000011604 | 162 | -33.1 | CHCHD4 |
| ssc-miR-615 | ENSSSCG00000011870 | 151 | -22.1 | PDIA5 |
| ssc-miR-615 | ENSSSCG00000012083 | 151 | -29.27 | RIPK4 |
| ssc-miR-615 | ENSSSCG00000012324 | 142 | -18.23 | IQSEC2 |
| ssc-miR-615 | ENSSSCG00000012655 | 157 | -34.47 | BCORL1 |
| ssc-miR-615 | ENSSSCG00000012743 | 143 | -19.75 | MTMR1 |
| ssc-miR-615 | ENSSSCG00000012785 | 148 | -21.08 | PDZD4 |
| ssc-miR-615 | ENSSSCG00000012795 | 172 | -34.69 | IRAK1 |
| ssc-miR-615 | ENSSSCG00000012845 | 165 | -30.95 | CEND1 |
| ssc-miR-615 | ENSSSCG00000012850 | 156 | -30.31 | DEAF1 |
| ssc-miR-615 | ENSSSCG00000012852 | 146 | -17.61 | CDHR5 |
| ssc-miR-615 | ENSSSCG00000012856 | 147 | -21.46 | NAP1L4 |
| ssc-miR-615 | ENSSSCG00000012874 | 165 | -31.97 | ORAOV1 |
| ssc-miR-615 | ENSSSCG00000012912 | 159 | -28.12 | TBC1D10C |
| ssc-miR-615 | ENSSSCG00000012947 | 152 | -25.65 | - |
| ssc-miR-615 | ENSSSCG00000012963 | 143 | -23.47 | SART1 |
| ssc-miR-615 | ENSSSCG00000013048 | 141 | -19.21 | C11orf84 |
| ssc-miR-615 | ENSSSCG00000013110 | 150 | -27.6 | TMEM109 |
| ssc-miR-615 | ENSSSCG00000013432 | 148 | -19.09 | MIDN |
| ssc-miR-615 | ENSSSCG00000013768 | 147 | -19.74 | - |
| ssc-miR-615 | ENSSSCG00000013883 | 144 | -21.41 | COLGALT1 |
| ssc-miR-615 | ENSSSCG00000025104 | 147 | -20.73 | - |

Table 1S (continued) The target genes prediction of miR-615

| miRNA名称miRNA name | 靶基因  Target genes | 评分Score | 能量值Energy | 基因名字  Gene name |
| --- | --- | --- | --- | --- |
| ssc-miR-615 | ENSSSCG00000005890 | 155 | -25.01 | ZNF7 |
| ssc-miR-615 | ENSSSCG00000005934 | 147 | -22.62 | TRAPPC9 |
| ssc-miR-615 | ENSSSCG00000006216 | 150 | -26.95 | TRIM55 |
| ssc-miR-615 | ENSSSCG00000006274 | 147 | -21.66 | PRKDC |
| ssc-miR-615 | ENSSSCG00000006510 | 162 | -31.03 | - |
| ssc-miR-615 | ENSSSCG00000006653 | 145 | -24.54 | ENSA |
| ssc-miR-615 | ENSSSCG00000006796 | 150 | -27.16 | CHI3L2 |
| ssc-miR-615 | ENSSSCG00000007289 | 159 | -23.17 | TRPC4AP |
| ssc-miR-615 | ENSSSCG00000007338 | 159 | -28.99 | - |
| ssc-miR-615 | ENSSSCG00000007369 | 142 | -17.11 | - |
| ssc-miR-615 | ENSSSCG00000007373 | 150 | -29.62 | GDAP1L1 |
| ssc-miR-615 | ENSSSCG00000007574 | 148 | -22.19 | SDK1 |
| ssc-miR-615 | ENSSSCG00000007656 | 148 | -24.27 | PVRIG |
| ssc-miR-615 | ENSSSCG00000007744 | 159 | -27.98 | PHKG1 |
| ssc-miR-615 | ENSSSCG00000007806 | 157 | -32.2 | RABEP2 |
| ssc-miR-615 | ENSSSCG00000007809 | 156 | -27.22 | SPNS1 |
| ssc-miR-615 | ENSSSCG00000007991 | 152 | -23.48 | WDR90 |
| ssc-miR-615 | ENSSSCG00000007992 | 146 | -23.58 | PIGQ |
| ssc-miR-615 | ENSSSCG00000007993 | 159 | -30.08 | CAPN15 |
| ssc-miR-615 | ENSSSCG00000008006 | 166 | -34.1 | FBXL16 |
| ssc-miR-615 | ENSSSCG00000008132 | 151 | -18.88 | STARD7 |
| ssc-miR-615 | ENSSSCG00000008238 | 151 | -21.37 | ELMOD3 |
| ssc-miR-615 | ENSSSCG00000009028 | 170 | -39.79 | - |
| ssc-miR-615 | ENSSSCG00000009584 | 156 | -23.31 | SEMA4D |
| ssc-miR-615 | ENSSSCG00000009629 | 159 | -24.52 | BIN3 |
| ssc-miR-615 | ENSSSCG00000009668 | 143 | -21.92 | CLU |
| ssc-miR-615 | ENSSSCG00000009875 | 149 | -22.65 | CFAP73 |
| ssc-miR-615 | ENSSSCG00000010046 | 147 | -25.68 | GNAZ |
| ssc-miR-615 | ENSSSCG00000010134 | 145 | -24.15 | TANGO2 |
| ssc-miR-615 | ENSSSCG00000026062 | 144 | -24.28 | - |

Table 1 (continued) The target genes prediction of miR-615

| miRNA名称miRNA name | 靶基因  Target genes | 评分Score | 能量值Energy | 基因名字  Gene name |
| --- | --- | --- | --- | --- |
| ssc-miR-615 | ENSSSCG00000024103 | 158 | -30.51 | - |
| ssc-miR-615 | ENSSSCG00000024382 | 156 | -21.95 | - |
| ssc-miR-615 | ENSSSCG00000024500 | 147 | -23.49 | - |
| ssc-miR-615 | ENSSSCG00000024545 | 149 | -22.01 | CHRM1 |
| ssc-miR-615 | ENSSSCG00000024907 | 148 | -22.36 | SDHAF1 |
| ssc-miR-615 | ENSSSCG00000024912 | 157 | -26.57 | WDR5 |
| ssc-miR-615 | ENSSSCG00000024929 | 175 | -34.6 | - |
| ssc-miR-615 | ENSSSCG00000024942 | 140 | -16.36 | - |
| ssc-miR-615 | ENSSSCG00000025047 | 151 | -29.11 | - |
| ssc-miR-615 | ENSSSCG00000002497 | 146 | -27.94 | TCL1B |
| ssc-miR-615 | ENSSSCG00000002759 | 143 | -21.05 | - |
| ssc-miR-615 | ENSSSCG00000002825 | 145 | -22.65 | - |
| ssc-miR-615 | ENSSSCG00000003042 | 151 | -22.88 | - |
| ssc-miR-615 | ENSSSCG00000003193 | 155 | -31 | TBC1D17 |
| ssc-miR-615 | ENSSSCG00000003222 | 166 | -38.73 | SYT3 |
| ssc-miR-615 | ENSSSCG00000003602 | 155 | -28.3 | PEF1 |
| ssc-miR-615 | ENSSSCG00000003976 | 150 | -22.38 | NFYC |
| ssc-miR-615 | ENSSSCG00000004465 | 149 | -30.81 | - |
| ssc-miR-615 | ENSSSCG00000005585 | 162 | -33.02 | - |
| ssc-miR-615 | ENSSSCG00000005841 | 141 | -16.15 | RABL6 |
| ssc-miR-615 | ENSSSCG00000029460 | 141 | -22.23 | RND2 |
