# Supplementary material for: miR-615 facilitates porcine epidemic diarrhea virus replication by targeting IRAK1 to inhibit type III interferon expression
Source: Front Microbiol. 2022 Dec 1;13:1071394. doi: 10.3389/fmicb.2022.1071394 (PMC9832332; doi:10.3389/fmicb.2022.1071394)
Supplement: Supplementary file 12 [file Table_12.DOCX]

| miRNA名称miRNA name | 靶基因  Target genes | 评分Score | 能量值Energy | 基因名字  Gene name |
| --- | --- | --- | --- | --- |
| ssc-miR-615 | ENSSSCG00000010254 | 149 | -24.14 | TACR2 |
| ssc-miR-615 | ENSSSCG00000010587 | 147 | -22.31 | TRIM8 |
| ssc-miR-615 | ENSSSCG00000010704 | 147 | -24.82 | LRFN1 |
| ssc-miR-615 | ENSSSCG00000010718 | 162 | -31.02 | CH242-129O9.1 |
| ssc-miR-615 | ENSSSCG00000011163 | 148 | -23.06 | - |
| ssc-miR-615 | ENSSSCG00000011322 | 151 | -25.45 | CCR1 |
| ssc-miR-615 | ENSSSCG00000011386 | 146 | -25.68 | MST1 |
| ssc-miR-615 | ENSSSCG00000011556 | 172 | -35.97 | IL17RE |
| ssc-miR-615 | ENSSSCG00000011604 | 162 | -33.1 | CHCHD4 |
| ssc-miR-615 | ENSSSCG00000011870 | 151 | -22.1 | PDIA5 |
| ssc-miR-615 | ENSSSCG00000012083 | 151 | -29.27 | RIPK4 |
| ssc-miR-615 | ENSSSCG00000012324 | 142 | -18.23 | IQSEC2 |
| ssc-miR-615 | ENSSSCG00000012655 | 157 | -34.47 | BCORL1 |
| ssc-miR-615 | ENSSSCG00000012743 | 143 | -19.75 | MTMR1 |
| ssc-miR-615 | ENSSSCG00000012785 | 148 | -21.08 | PDZD4 |
| ssc-miR-615 | ENSSSCG00000012795 | 172 | -34.69 | IRAK1 |
| ssc-miR-615 | ENSSSCG00000012845 | 165 | -30.95 | CEND1 |
| ssc-miR-615 | ENSSSCG00000012850 | 156 | -30.31 | DEAF1 |
| ssc-miR-615 | ENSSSCG00000012852 | 146 | -17.61 | CDHR5 |
| ssc-miR-615 | ENSSSCG00000012856 | 147 | -21.46 | NAP1L4 |
| ssc-miR-615 | ENSSSCG00000012874 | 165 | -31.97 | ORAOV1 |
| ssc-miR-615 | ENSSSCG00000012912 | 159 | -28.12 | TBC1D10C |
| ssc-miR-615 | ENSSSCG00000012947 | 152 | -25.65 | - |
| ssc-miR-615 | ENSSSCG00000012963 | 143 | -23.47 | SART1 |
| ssc-miR-615 | ENSSSCG00000013048 | 141 | -19.21 | C11orf84 |
| ssc-miR-615 | ENSSSCG00000013110 | 150 | -27.6 | TMEM109 |
| ssc-miR-615 | ENSSSCG00000013432 | 148 | -19.09 | MIDN |
| ssc-miR-615 | ENSSSCG00000013768 | 147 | -19.74 | - |
| ssc-miR-615 | ENSSSCG00000013883 | 144 | -21.41 | COLGALT1 |
| ssc-miR-615 | ENSSSCG00000025104 | 147 | -20.73 | - |
